# *In Vitro* Testing of Scaffolds for Mesenchymal Stem Cell-Based Meniscus Tissue Engineering—Introducing a New Biocompatibility Scoring System

**DOI:** 10.3390/ma9040276

**Published:** 2016-04-07

**Authors:** Felix P. Achatz, Richard Kujat, Christian G. Pfeifer, Matthias Koch, Michael Nerlich, Peter Angele, Johannes Zellner

**Affiliations:** 1 Department of Trauma Surgery, University Medical Centre Regensburg; Franz Josef Strauss Allee 11, 93053 Regensburg, Germany; felix.achatz@googlemail.com (F.P.A.); richard.kujat@ukr.de (R.K.); christian.pfeifer@ukr.de (C.G.P.); matthias.koch@ukr.de (M.K.); michael.nerlich@ukr.de (M.N.); peter.angele@ukr.de (P.A.); 2 Sporthopaedicum Regensburg, Hildegard von Bingen Strasse 1, 93053 Regensburg, Germany

**Keywords:** meniscus, polyurethane scaffold, composite scaffold, hyaluronic acid, collagen, gelatin, chondrogenesis, human mesenchymal stem cells, biocompatibility

## Abstract

A combination of mesenchymal stem cells (MSCs) and scaffolds seems to be a promising approach for meniscus repair. To facilitate the search for an appropriate scaffold material a reliable and objective *in vitro* testing system is essential. This paper introduces a new scoring for this purpose and analyzes a hyaluronic acid (HA) gelatin composite scaffold and a polyurethane scaffold in combination with MSCs for tissue engineering of meniscus. The pore quality and interconnectivity of pores of a HA gelatin composite scaffold and a polyurethane scaffold were analyzed by surface photography and Berliner-Blau-BSA-solution vacuum filling. Further the two scaffold materials were vacuum-filled with human MSCs and analyzed by histology and immunohistochemistry after 21 days in chondrogenic media to determine cell distribution and cell survival as well as proteoglycan production, collagen type I and II content. The polyurethane scaffold showed better results than the hyaluronic acid gelatin composite scaffold, with signs of central necrosis in the HA gelatin composite scaffolds. The polyurethane scaffold showed good porosity, excellent pore interconnectivity, good cell distribution and cell survival, as well as an extensive content of proteoglycans and collagen type II. The polyurethane scaffold seems to be a promising biomaterial for a mesenchymal stem cell-based tissue engineering approach for meniscal repair. The new score could be applied as a new standard for *in vitro* scaffold testing.

## 1. Introduction

Lesions of the meniscus are amongst the most frequent injuries in orthopedic surgery [1]. In many cases partial meniscectomy has to be performed due to the poor healing capacity of the avascular part of the meniscus [2]. This however predisposes for osteoarthritic changes of the affected knee joint [2,3,4]. Knee menisci are essential in providing joint stability [5], lubrication [6], proprioception [7], force transmission [8], and shock absorption [9]. Additionally, partial meniscectomy causes severe changes in the biomechanics of the knee joint that are directly proportional to the amount of lost tissue [10] resulting in drastically increased contact pressure to the surrounding cartilage [11]. Therefore, it is essential to restore as much meniscus substance as possible. While successful repair strategies for lesions in the vascular part of the meniscus have been developed, there is still no sufficient therapy for lesions in the avascular part [12]. Currently two scaffolds are used in clinic in a cell-free approach for meniscus repair: CMI^®^, a collagen scaffold, and Actifit^®^, a polyurethane scaffold. Both scaffolds have shown promising results [1,13,14,15], however, as of yet no large randomized studies with control groups have been published. Recently, mesenchymal stem cells have been a focus of attention in newly developed approaches for meniscus repair [15]. As these cells have both the potential to differentiate into fibro chondrocytes and the ability to secrete repair promoting growth factors they seem an ideal tool for meniscus repair [16]. Preclinical studies have already shown the repair potential of mesenchymal stem cells in combination with a scaffold in the treatment of relatively small tears and punch defects in the avascular zone of the meniscus [17,18,19]. A recent review article highlights the capability of cells to enhance meniscus repair, however, it remains unclear which biomaterial is best suited for this purpose [20]. With new scaffold materials evolving, costly animal experiments have to be conducted before putting them into clinical trials. To save time, resources and animals, a reliable *in vitro* testing system is needed to single out promising materials before going into *in vivo* experiments. The object of this paper is to thoroughly test two biomaterials for their suitability to be used in a mesenchymal stem cell based approach for tissue engineered meniscus. Both to rank our own results as well as to introduce a new standard for biomaterial testing we created a scoring system. We tested the polyurethane scaffold Actifit^®^ that has already shown promising results in clinical trials [1], as well as a hyaluronic acid gelatin composite scaffold that has shown good results in several *in vivo* experiments [19].

## 2. Materials and Methods

### 2.1. Production of Composite Scaffolds

Hyaluronic acid gelatin composite scaffolds were produced as described elsewhere [21,22]. Briefly, the material contained 70% hyaluronic acid (commercially available product, Jaloskin^®^, Fidia Advances Biopolymers, Abano Terme, Italy) and 30% gelatin (hydrolyzed bovine collagen, Sigma, Seelze, Germany). To obtain a porous material solvent casting particulate leaching technique was applied. Both components were solved, mixed, and air dried after the addition of NaCl-chrystals. Salt was washed out with water and the material was then dried in vacuum. Pore size was 350–450 μm. The product was cut in smaller parts and then sterilized with 25 kGy beta rays (Beta-Gamma-Service GmbH, Saal, Germany).

### 2.2. Polyurethane Scaffolds

The commercially available polyurethane scaffold Actifit^®^ (Orteq, London, UK) was used for this study. The product was cut in smaller parts and then sterilized with 25 kGy beta rays (Beta-Gamma-Service GmbH, Saal, Germany).

### 2.3. Macroscopic Assessment of Scaffold Pore Structure

For each biomaterial a whole biomaterial meniscus was cut into 2 mm thick slices whose surfaces were subsequently photographed. Using highly-magnified and printed photographs, the quality the pore structure was assessed by two blinded and experienced scorers. Scorers conducted this by using the naked eye to get a quick and simple overview, enabling a semi-quantitative analysis.

### 2.4. Interconnectivity of Scaffold Pores

For each biomaterial two 2 mm thick slices were filled with a Berliner-Blau-BSA-solution containing 10% Berliner Blau and 10% BSA in distilled water. Filling was conducted by vacuum as described below. Filled scaffolds were fixated overnight in phosphate buffer 0.1 M with 4% paraformaldehyde and 1% glutaraldehyde and embedded in TissueTek^®^ (Sakura Finetek Japan Co., Tokyo, Japan) the following day using liquid nitrogen. Blocks were cut in the cryotome and their surfaces were repeatedly photographed after each 100 μm cut in order to create a representative series of images. The Berliner-Blau-BSA-solution was only found in pores that had indirect access to the surface of the scaffold and were, thus, interconnected. A qualitative assessment of the interconnectivity was conducted by two blinded and experienced scorers. Scorers used the naked eye to semi-quantitatively determine the percentage of interconnected pores, which is equivalent to the percentage of the scaffold area that shows the typical Berliner-Blau color.

### 2.5. Isolation and Culture of Human Mesenchymal Stem Cells

After approval by the local ethical committee and patients’ informed consent, bone marrow-derived MSCs were acquired from patients with surgery that included harvest from the iliac crest.

Mononuclear cells were layered over the Ficoll-Paque (GE Healthcare Bio-Sciences, Marlborough, MA, USA) density-gradient. A heparinized syringe was used to aspirate the layer with MSCs, Dulbecco’s modified Eagle’s medium (DMEM), low glucose concentration (5%), with 10% fetal bovine serum, 1% penicillin, and 1% Hepes buffer was added and 2 × 10^6^ nucleated cells were plated per T75 cell culture dish. The adherent cells expanded quickly and media was changed twice a week until reaching of 80% confluence.

### 2.6. In Vitro Chondrogenesis

Upon reaching 80% confluence mesenchymal stem cells were trypsinized, counted, washed, and re-suspended in a chemically-defined chondrogenic medium to final concentration of 2 × 10^4^ cells/μL. As previously described by Angele *et al.* [23] chondrogenic media contained DMEM (high glucose), 200 μM ascorbic acid-2 phosphate, 1% insulin-transferrin-sodium selenite media supplement (ITS) (both from Sigma, Taufkirchen, Germany), 1 mM pyruvate, 100 nM dexamethasone, and 10 ng/mL transforming growth factor ß1(TGFß1) (R and D systems, Wiesbaden, Germany).

Cylindric (diameter: 5 mm, height: 2 mm) scaffold parts were then loaded with 50 μL of the cell suspension (1 × 10^6^ cells) per scaffold part. From each biomaterial, 6–12 scaffolds were loaded. Loading of the scaffolds was achieved using a rotary valve vacuum pump (Vacuubrand GmbH, Wertheim, Germany). Both scaffold and cell suspension were placed into cylindrical tubes. Vacuum was then applied for 10 s, followed by a brief ventilation. This was repeated 10 times. The strength of the applied vacuum was adjusted manually to an extent that showed moderate foam generation in the media.

The loaded scaffolds were then incubated at 37 °C for 1 h and five scaffolds of each biomaterial were then kept in chondrogenic media for 21 days under normoxic conditions, media was changed three times a week. Per biomaterial, one scaffold was fixated on the next day, serving as a loading control.

### 2.7. Histology

The scaffolds from the *in vitro* differentiation were fixed in a 1 M phosphate buffer solution containing 4% paraformaldehyde, embedded in Tissue-Tek OCT (Sakura Finetek, Tokyo, Japan) and frozen in liquid nitrogen. All samples were cut in 10 μm sections and every 10th of them was stained with dimethylmethylen blue (DMMB). Two blinded scorers analyzed the sections according to the proposed scoring system. This was conducted by the naked eye to assess semi-quantitative differences. Both scorers were experienced in analyzing histological cartilage sections.

To evaluate the cell viability we searched for secure histological signs of cell death, such as blurred cell nucleus borders and loss of adherence. Cell distribution was assessed by comparing the number of cell-populated pores to the number of cell-free pores. The content of proteoglycan was measured by determining the percentage of metachromatic extracellular matrix in scaffold pores.

### 2.8. Immunohistochemistry

Sections were washed followed by a 15 min digestion with 0.1% pepsin at pH 3.5 for a facilitated antibody access to the target epitopes. Type I and II collagen were immunolocalized by the immunoperoxidase ABC technique (Vector, Burlingame, CA, USA). As primary antibodies anti-collagen II (clone II-4C11; Calbiochem Merck, Schwalbach, Germany) and monoclonal CD31 mouse anti-rabbit antibodies (clone JC-70A IgG1 light chain type kappa; Abcam, Cambridge, UK) were used. After staining with biotin conjugated polyclonal goat anti mouse IgG secondary antibody (Jackson, West Grove, PA, USA), positive signals were visualized by nickel- and cobalt-enhanced 3,3′-diaminobenzidine (DAB). Two blinded scorers analyzed the sections semi-quantitatively according to the proposed scoring system. Percentage of content of collagen I or collagen II was determined by comparing DMMB stained slides with immunohistochemically stained slides.

### 2.9. Score

To our knowledge as of yet there has not been described a score for standardized *in vitro* analysis and comparison of newly developed biomaterial for stem cell-based meniscus repair. Therefore, we propose the following score.

For scoring item 1, magnified photographs of the surface of the biomaterial slices were analyzed. Berliner-Blau-solution filled biomaterial slices were analyzed for scoring item 2.

For scoring items 3–7, five to ten scaffolds per biomaterial were analyzed after being filled with MSCs and 21 days of differentiation in chondrogenic media.

Further details about assessing the scoring values’ different parameters are outlined in the above paragraphs.

Each of this scaffolds was assessed on its own for each scoring item and the average scoring value was noted as a whole number value in the score. Scoring values are displayed in Table 1.

That way seven individual scoring subgroups were formed, each received a scoring value ranging from 0 (bad) to 3 (ideal). The values of these items were summed up, consequently reaching a combined score from 0 (material unsuitable for meniscus repair) to 21 (promising material for MSC-based meniscus repair). Two experienced blinded scorers conducted the data collection. A high internal consistency has been attributed to this scoring system by a statistician from the Center of Clinical Studies of the University of Regensburg, thus making it legitimate to sum up the single item scores.

### 2.10. Statistical Analysis

To determine whether data followed a Gaussian distribution a Kolmogorov–Smirnov test was conducted. For comparison of non-normal distributed data the Mann–Whitney U-test was used. A probability value of less than 0.05 was set as the level of statistical significance for all evaluations.

## 3. Results

We conducted a thorough test of two biomaterials, one being a hyaluronic acid gelatin composite scaffold developed in our own working group, the other one being the commercially available polyurethane based product Actifit^®^ which is already in clinical use for cell-free meniscal replacement after subtotal loss of meniscus substance.

### 3.1. Macroscopical Assessment of Scaffold Pore Structure

**Figure 1:**
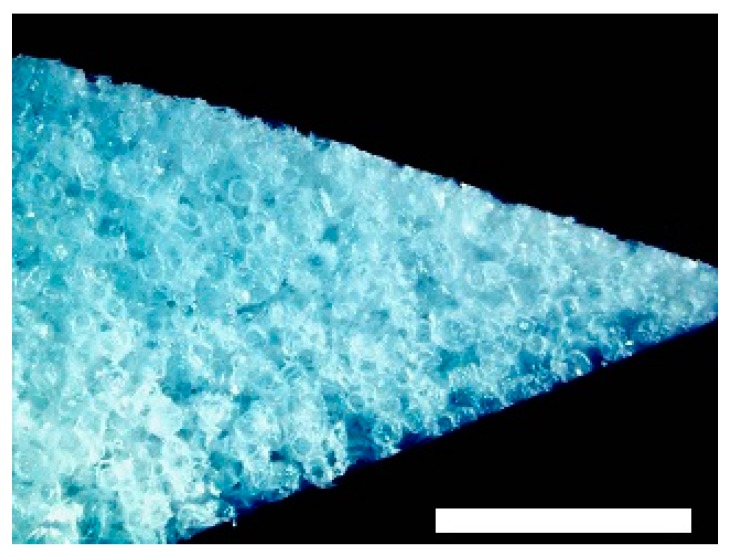
Macroscopic enhanced image of a hyaluronic acid gelatin composite scaffold. Scale bar = 1 mm.

**Figure 2:**
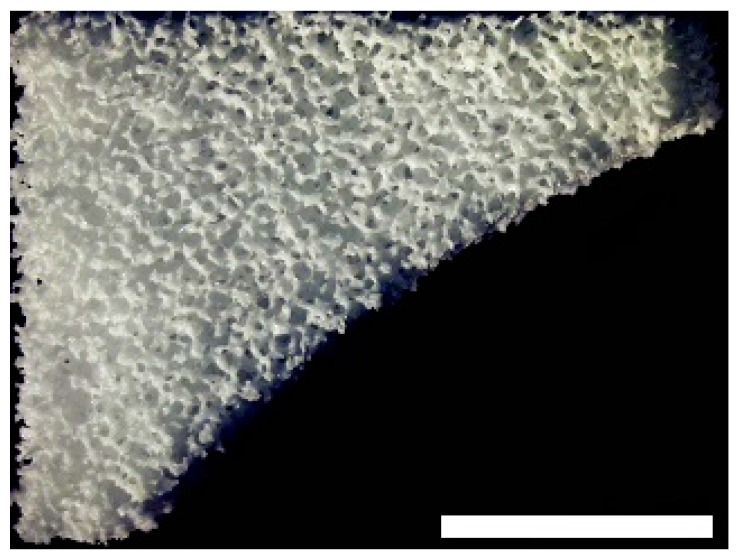
Macroscopic enhanced image of an Actifit^®^ scaffold. Scale bar = 1 mm.

The biomaterials were cut into 2 mm thick slices to assess the quality of the porosity. Both the hyaluronic acid gelatin composite scaffold as well as the Actifit^®^ scaffold showed an even distribution of pores that varied less than 50% in size (Figure 1 and Figure 2).

### 3.2. Interconnectivity of Scaffold Pores

**Figure 3:**
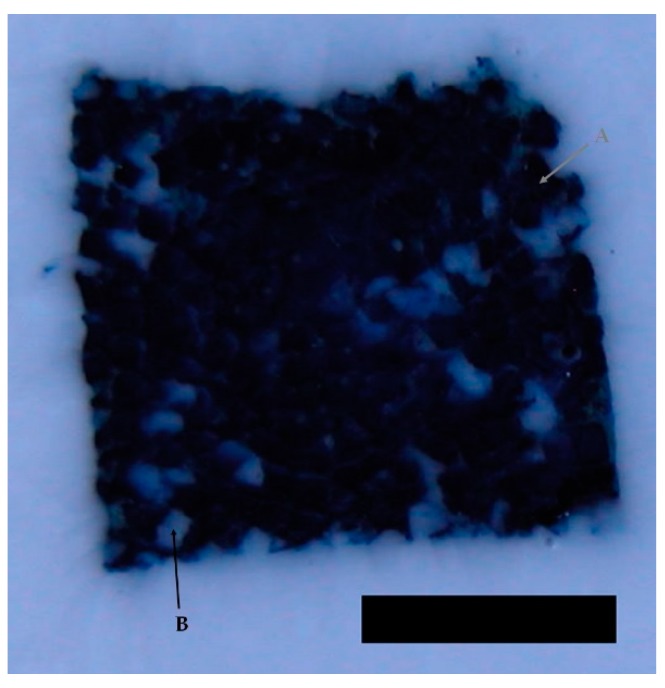
Illustration of pore interconnectivity in a hyaluronic acid gelatin composite scaffold. Black areas represent accessible pores whereas white areas signify secluded pores. Arrow A points to an interconnected, accessible pore. Arrow B points to a non-interconnected, secluded pore. Most of the scaffold’s area is shows a black color, thus proving excellent pore interconnectivity. Magnification bar = 1 mm.

**Figure 4:**
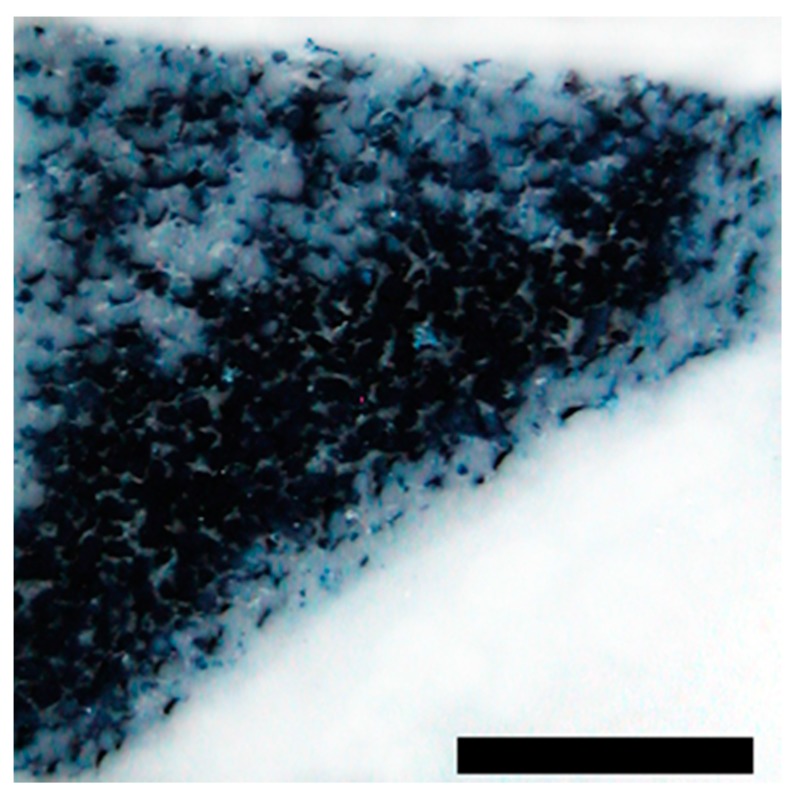
Illustration of pore interconnectivity in an Actifit^®^ scaffold. Black areas represent accessible pores whereas white areas signify secluded pores. A major part of the scaffold’s area is shows a black color, thus proving good pore interconnectivity Magnification bar = 1 mm.

To assess the interconnectivity of the scaffolds’ pores the scaffolds were filled with a Berliner-Blau-BSA-solution that could only penetrate into interconnected pores. The hyaluronic acid gelatin composite scaffold showed a very strong interconnectivity of pores with almost no pores remaining unfilled. The Actifit^®^ scaffold showed good interconnectivity of pores in the center of the scaffold, however, many pores in the scaffold’s periphery remained unfilled (Figure 3 and Figure 4).

### 3.3. In Vitro Chondrogenesis

To analyze the biocompatibility of the materials an *in vitro* experiment was conducted. Per biomaterial six scaffold cylinders, 2 mm in height, and 5 mm in diameter were filled with human MSCs and kept in chondrogenic media for 21 days.

**Figure 5:**
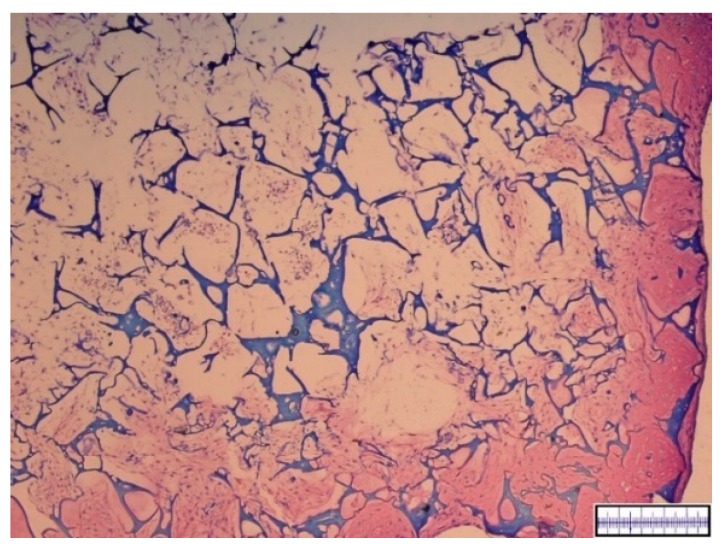
Representative histological slide of a hyaluronic acid gelatin composite scaffold after 21 days of *in vitro* chondrogenesis with visible central necrosis. DMMB staining. Proteoglycan-rich extracellular matrix appears red, scaffold parts appear blue. Magnification bar = 500 μm.

**Figure 6:**
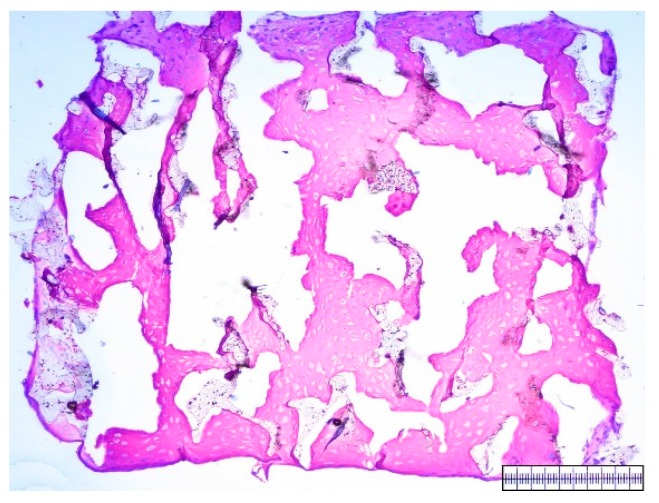
Representative histological slide of an Actifit^®^ scaffold after 21 days of *in vitro* chondrogenesis. DMMB staining. Proteoglycan-rich extracellular matrix appears red, scaffold parts appear grey and porous. Magnification bar = 500 μm.

Upon harvest the hyaluronic acid gelatin composite scaffolds appeared instable, sensitive to touch with obvious signs of degradation. DMMB staining showed an initially excellent cell distribution with more than 75% of pores being populated by cells. However, after 21 days the survival rate of cells was unsatisfying, with an approximate average of 50% percent of cells showing signs of necrosis, especially in the central parts of the scaffolds. Accordingly, only the peripheral pores of the scaffolds were extensively filled with proteoglycans, whereas the central parts remained fairly empty. Figure 5 and Figure 6 show representative DMMB-stained slices.

Immunohistochemistry showed high levels of both collagen I and II in peripheral parts and almost no collagen I or II in the central parts of the scaffolds. 

**Figure 7:**
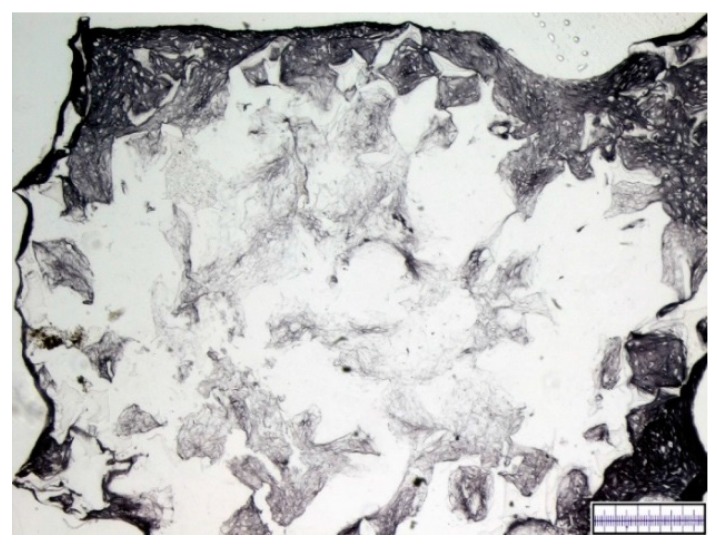
Representative slide of a hyaluronic acid gelatin composite scaffold after 21 days of *in vitro* chondrogenesis. Collagen type I immunohistochemistry. Collagen type I-rich areas appear black. Magnification bar = 500 μm.

**Figure 8:**
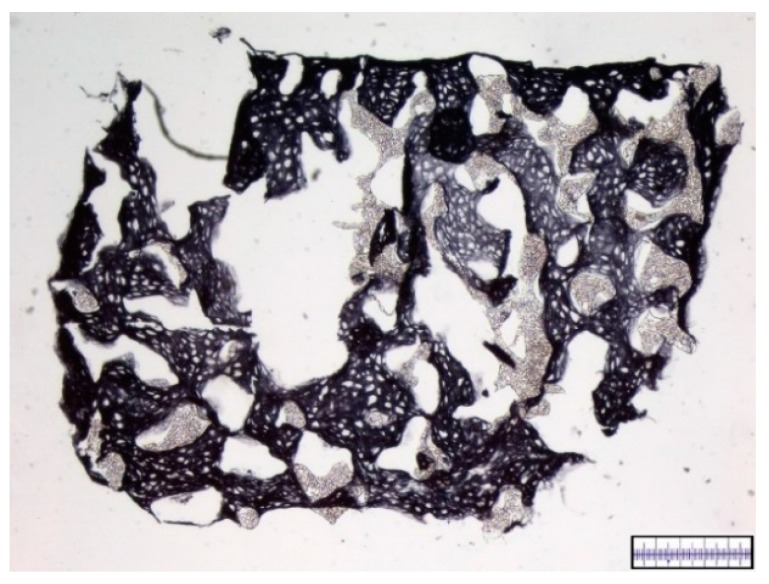
Representative slide of an Actifit^®^ scaffold after 21 days of *in vitro* chondrogenesis. Collagen type I immunohistochemistry. Collagen type I-rich areas appear black. Magnification bar = 500 μm.

**Figure 9:**
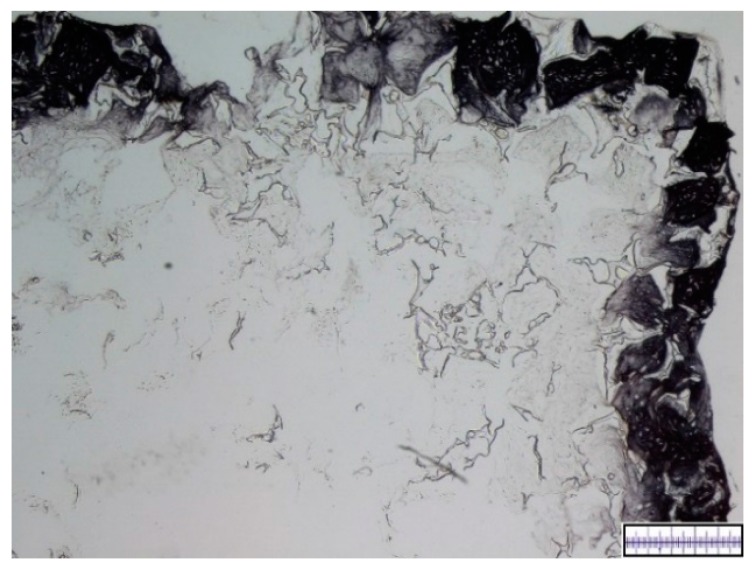
Representative slide of a hyaluronic acid gelatin composite scaffold after 21 days of *in vitro* chondrogenesis. Collagen type II immunohistochemistry. Collagen type II-rich areas appear black. Magnification bar = 500 μm.

**Figure 10:**
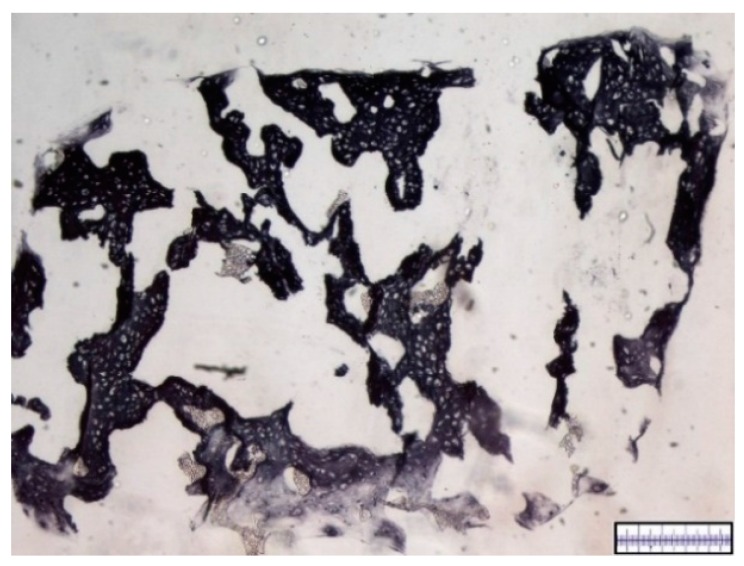
Representative slide of an Actifit^®^ scaffold after 21 days of *in vitro* chondrogenesis. Collagen type II immunohistochemistry. Collagen type II-rich areas appear black. Magnification bar = 500 μm.

The Actifit^®^ scaffolds appeared stable upon harvest with no obvious signs of degradation. Initial cell distribution was excellent with more than 75% of pores being cell-populated. No signs of necrosis were observed. Throughout the whole scaffolds extensive production of proteoglycans was noted. Immunohistochemistry showed high levels of collagen type II and moderate levels of collagen type I content. Figure 7 and Figure 8 show representatives slices with collagen type I immunohistochemistry. Collagen type II immunohistochemistry is displayed in Figure 9 and Figure 10.

### 3.4. Scoring

**Figure 11:**
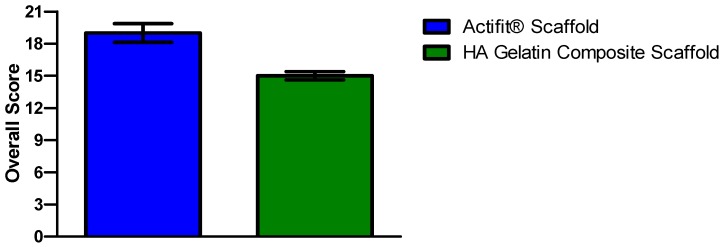
Overall Scoring Results of the different scaffolds. Error bars indicate 95% confidence interval.

In our scoring system scaffolds receive an overall score ranging from 0 (=not suitable) to 21 (=very promising). Both hyaluronic acid gelatin composite scaffold and Actifit^®^ received promising results, however, Actifit^®^ still obtained a clearly higher total score of 19 points compared to the hyaluronic acid gelatin composite scaffold’s 15 points (Figure 11).

**Figure 12:**
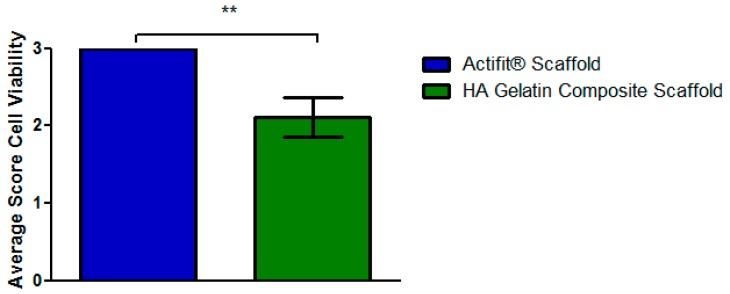
Average scoring results of the different scaffolds for single score item viability of cells. Error bars indicating 95% confidence interval. Scoring item was assessed in a semi-quantitative manner. Cell viability was statistically significant better (Mann–Whitney-U test, ** = *p* < 0.05) in the Actifit^®^ scaffolds compared to the HA (hyaluronic acid) gelatin composite scaffolds.

The hyaluronic acid gelatin composite scaffold received statistically significant less points (*p* < 0.05) for cell survival compared to Actifit^®^, because necrosis took place in the central parts of the hyaluronic acid gelatin composite scaffolds. The diminished number of vital cells consequently produced fewer proteoglycans and collagen II, thus leading to further point losses in these categories. The scoring results for cell viability are displayed in Figure 12.

## 4. Discussion

The main purpose of this paper is to introduce a new scoring system that can be universally applied for *in vitro* testing of biomaterials for scaffold-based cell-enhanced meniscus repair. To demonstrate the execution and validity of our score we used it to compare two scaffolds that have been previously used both in an *in vitro* setting, an *in vivo* setting in an animal model [15,18,19,24]. One of the scaffolds is already clinically used in a cell-free approach [1].

We thoroughly analyzed two biomaterial scaffolds regarding their capacity for mesenchymal stem cell-based meniscus repair: the polyurethane scaffold Actifit^®^ and a hyaluronic acid gelatin composite scaffold developed in our own working group. To objectify our results as well as to facilitate and standardize further testing we introduced a new point-based scoring system for the *in vitro* testing of biomaterial for meniscus repair.

In our test both scaffolds showed good results; however, Actifit^®^ received a clearly better overall score. The hyaluronic acid gelatin composite scaffold showed excellent porosity, however, cell survival was limited with necrosis taking place in the central parts of the scaffolds. Consequently less proteoglycans and collagens were produced. Actifit^®^ received excellent results throughout our test, this seems to support the argument that this scaffold should be further investigated in *in vivo* experiments and clinical trials.

In existing clinical trials [1,15,25] as well as in *in vivo* experiments [26,27] Actifit^®^ showed good results with statistically significant improvements in clinical outcome as well as improved macroscopic and histologic meniscus healing. The hyaluronic acid gelatin composite scaffold showed promising results in several *in vivo* studies [18,19,23]. These good results of both scaffolds in *in vivo* experiments and clinical trials are concordant with the good results these scaffolds received in our *in vitro* test. This seems to prove the assumption that our new score can be a reliable tool to determine which biomaterials are worth being further tested in *in vivo* experiments and subsequent clinical trials. Using our new *in vitro* testing system research resources could be saved by saving money, time and animal lives. Furthermore our score can be used to standardize biomaterial testing, thus making it possible to objectively compare results amongst different working groups.

As described in the next paragraphs, our score incorporates the assessment of the most important qualities of scaffolds for meniscus repair. According to several authors [28,29] scaffolds should have the following qualities: biocompatibility, open pores to enable tissue ingrowth and properties for cell adhesion.

Especially the quality of porosity is an essential feature for successful tissue engineering of the meniscus with mesenchymal stem cell-filled scaffolds [30].

Therefore, our scoring system contains three single scoring items that are directly or indirectly connected with the quality of the scaffold porosity: the quality of pore structure, the interconnectivity of pores and the cell distribution in the scaffolds.

As mentioned above, biocompatibility is a vital feature for a successful biomaterial. Our testing system thoroughly assesses the biocompatibility of the scaffolds with the scoring items of the *in vitro* experiment*: viability of cells* and *production of proteoglycans*.

Furthermore it is important for the cells inside the scaffolds to not merely produce scar tissue but to produce mechanically stable meniscus-like tissue. The dry mass of the human meniscus mainly consists of collagen [31] and our chondrogenesis model is specifically set to enable production of collagen II. Consequently, our score rewards high contents of collagen type II as this also shows the successful differentiation of the MSCs. In contrast, collagen type I production rather hints to undifferentiated scar tissue, which is why a low content of collagen type I is rewarded with a high score in our testing system.

A recent review article gives an overview about the currently used scoring systems for meniscus repair [32]. While none of these scoring systems are intended for the *in vitro* evaluation of biomaterial, they nonetheless frequently analyze certain key tissue characteristics that are also assessed in our score. These central tissue characteristics include cellularity, collagen formation, and proteoglycan content. The aspect of scaffold porosity has mostly been neglected in the described scoring systems. However, we consider porosity to be a central aspect for scaffolds and, therefore, made it a big part of our score.

As demonstrated, our score covers the most important properties of a good biomaterial scaffold for meniscus repair and therefore qualifies as a highly effective tool with a very accurate prediction value for the assessment of new biomaterials. Our score assesses differences in key characteristics that are vital for any type of scaffold; consequently, our score can be applied for testing any kind of biomaterial that is intended for cell-enhanced meniscus repair. Our scoring system offers a thorough, but easily reproducible, seven step evaluation that can help investigators to both objectify their results and to compare the qualities of different biomaterials.

In biomaterial research a variety of animal types has been used, amongst others: rabbits, dogs, and sheep. As our score relies on standardized *in vitro* conditions we eliminate the variability of results that is involved when using different types of animals for *in vivo* studies.

Mesenchymal stem cells have been a focus of research as a source for tissue engineering, which is why we used mesenchymal stem cells in our experiments. However, our scoring system could also be used for a combination of scaffold with different cell types.

There are some limitations to our study, which we purposely tolerate for different reasons. While biomechanics are an essential property of any biomaterial [33] our score does not examine it. Biomechanics and biocompatibility should be assessed separately, as it facilitates identification of parameters that have to be adjusted. Furthermore, a thorough test regime for the functional and biomechanical analysis of scaffolds has already been described by Maher *et al* [26]. This test regime can easily be combined with our scoring system, thus creating a complete evaluation of a biomaterial. Another limitation of our study might be the relatively low-key use of quantification, software, and special viability staining, as well as the relying on naked eye assessment. However, we wanted to create a simple score that can quickly and easily be applied by any investigator without having to purchase expensive dyes or software. Experienced meniscus investigators are capable of a reliable and objective naked eye examination that gives valuable qualitative information. Our score possesses the advantage to function not only with a relatively simple approach like presented in this study, but also with a more quantified approach, as single scoring items could also be assessed with quantification software or assays, if this is desired. For these reasons and despite its limitations our scoring system presents a valuable novel tool for the evaluation of scaffolds. Our score gives a quick overview whether a newly developed bio material is worth to proceed to further research steps for example in an *in vivo* setting.

Given the good results the Actifit^®^ scaffold reached in our experiments it qualifies for mesenchymal stem cell-based meniscus repair and should be further investigated in *in vivo* experiments and clinical trials.

As mentioned above, cell-free scaffolds are currently used in clinics with promising results, however, this approach could possibly be further enhanced by the addition of mesenchymal stem cells. The ideal cell for cell-based meniscus repair should be autologous, obtainable in sufficient numbers in a minimal-invasive procedure and it has to possess the ability to be expanded in tissue culture and to produce stable fibrocartilaginous extracellular matrix [34]. These qualities are met by MSCs: they are easily obtainable via bone marrow puncture [35], can be expanded in tissue culture without losing their stem cell characteristics [36], and have shown the ability to produce fibrocartilaginous extracellular matrix [18].

Several studies have shown a statistically significant benefit that resulted by loading a scaffold with MSCs compared to cell-free scaffolds [18,19]. This benefit seems to originate from a dual role of the MSCs in meniscus repair [17]. Firstly, MSCs could directly heal meniscus lesions, as they can differentiate into fibro chondrocytes [37]. Secondly, MSCs could indirectly enhance meniscus healing via secretion of bioactive substances [38] that promote self-healing. Furthermore, MSCs possess immunoregulatory properties and could, thus, prevent tissue destruction and scar formation in injured tissue [38]. Consequently, the use of MSCs to enhance scaffold-based meniscus repair seems to be a promising approach.

## 5. Conclusions

The new score we developed could be applied as a new standard for *in vitro* scaffold testing of different biomaterials and, thus, save precious financial resources, as well as research animals and time.

The polyurethane scaffold showed excellent results in our *in vitro* test and seems to be a promising biomaterial for tissue engineering with MSCs. *In vivo* studies with a mesenchymal stem cell-filled Actifit^®^ scaffold will determine whether this approach can be applied in clinics.

## Figures and Tables

**Table 1:** Scoring system for analysis of biomaterials used for meniscal substitution or tissue engineering of the meniscus.

Scoring Value	0	1	2	3
Quality of pore structure	Pore size varies more than 200% compared to the average pore size	Pore size varies 100%–200% compared to the average pore size	Pore size varies 50%–100% compared to the average pore size	Pore size varies less than 50% compared to the average pore size
Interconnectivity of pores, percentage of interconnect pores	0%	1%–25%	26%–75%	More than 75%
Cell distribution in scaffolds (*in vitro*), percentage of cell-populated pores	0%	1%–25%	26%–75%	More than 75%
Cell viability in scaffolds (*in vitro*)	0%	1%–25%	26%–75%	More than 75%
Content of Proteoglycan (*in vitro*)	No staining for proteoglycan	<25%	25%–75%	>75%
Percentage of Collagen I compared to total amount of extracellular matrix	More than 75%	25%–75%	<25%	No staining for collagen I
Percentage of Collagen II content compared to total amount of extracellular matrix	No staining for collagen II	<25%	25%–75%	>75%

## Abbreviations

The following abbreviations are used in this manuscript:

BSA: bovine albumin solution
DMMB: dimethylemethylene blue
MSCs: mesenchymal stem cells
HA: hyaluronic acid
TGF-beta: transforming growth factor beta
